# Urinary incontinence and the association with depression, stress, and self-esteem in older Korean Women

**DOI:** 10.1038/s41598-021-88740-4

**Published:** 2021-04-27

**Authors:** Hoo-yeon Lee, Yumie Rhee, Kui Son Choi

**Affiliations:** 1 Department of Social Medicine, College of Medicine, Dankook University, Chungnam, South Korea; 2 Division of Endocrinology, Department of Internal Medicine, Yonsei University College of Medicine, Seoul, South Korea; 3 Graduate School of Cancer Science and Policy, National Cancer Center, Goyang-si, South Korea

**Keywords:** Health care, Risk factors, Urology

## Abstract

The objectives were to investigate the prevalence of urinary incontinence (UI) and its relationships with depression, stress, and self-esteem in older Korean women from the Korean Study of Women’s Health Related Issues (K-Stori), a nationally representative cross-sectional survey. A total of 3000 women between 65 and 79 years were the final study subjects. We applied multiple linear regression models to analyze associations with depression, stress, and self-esteem levels in relation to UI types. Types of urinary incontinence included stress, urge, and mixed UI. UI affects at least one in two older Korean women (52.2%). The prevalences of SUI, UUI, and MUI were 45.7%, 39.6%, and 33.1%, respectively. UI was found to be adversely associated with depression, stress, and self-esteem: women with UI reported significantly higher levels of depression and stress and lower levels of self-esteem than those without UI. Women with MUI reported significantly greater impairment than the women with SUI or UUI. Our results provide an evidence base for the evaluation of mental health in older women with incontinence. The prioritization of UI detection and the identification of psychological factors may help improve the diagnosis and management of UI and potentially yield significant economic and psychosocial benefits.

## Introduction

Urinary incontinence (UI) is defined as the involuntary leakage of urine. Although this condition can occur in adults of all ages, a large body of research has shown that the prevalence of UI increases with age^1^. With a significant impact on morbidity and quality of life, UI has been shown to affect an older person’s emotional wellbeing, social function, and general health and to be associated with poorer mental health, including depression, low self-esteem, anxiety, and cognitive impairment^1–4^. Meanwhile, research has indicated that poorer mental health can affect the course and outcomes of UI^1^: Among women who reported experiencing incontinence, about half (48%) expressed concerns about their symptoms getting worse in the future. Forty percent reported embarrassment regarding urinary leakage, and one in three (32%) said they worried about incontinence-related odor.

Urinary incontinence is a broad term and may include stress, urge, and mixed UI, as well as UI of other types. Urge urinary incontinence (UUI) is defined as involuntary urine leakage associated with urgency. Stress urinary incontinence (SUI) is defined as involuntary urine leakage associated with specific activities (e.g., sneezing and coughing). Mixed urinary incontinence (MUI) includes features of both UUI and SUI^5^. The reported risk factors of UI include age, number of childbirths, previous hysterectomy, obesity, and chronic disease^3,4^.

With the rapid aging of societies, UI is a growing worldwide problem. In several large studies of U.S. women aged 50 years and older, approximately one-third to two-thirds of women reported UI^2^. UI is estimated to affect 30% to 40% of women in the UK^6^. In a previous study for Korea, UI was reported by 40.8% of Korean female adults aged 30–79 years; the prevalences of SUI, UUI, and MUI were 22.9, 3.1, and 14.9%^7^. Across studies, the reported prevalences for UI of any subtype in adult women vary greatly (5–72%). This enormous variation between studies could be due to cultural differences in the perception of urinary incontinence, willingness to report urinary incontinence, methodological differences, and differences in case definitions^8^.

Although UI is not a life-threatening condition, the severe social impairment associated therewith can negatively affect the lives of patients. This suggests that UI cannot be adequately treated without consideration of a patient's overall quality of life^9^. However, studies on the impact of urinary incontinence on quality of life in older Korean women are lacking, despite numerous clinical observations supporting its negative influence^10^. The purposes of this study were to investigate the prevalence of UI and to compare the prevalence of psychological symptoms (depression, stress, and self-esteem) according to the presence or absence of UI.

## Results

Table 1 describes the sociodemographic characteristics and mental health of women aged 65–79 years. The average age of the study population was 71.8 years (standard deviation [SD], ± 4.3) years. The percentage of obese women was 31.4%. The majority of the study population (72.6%) reported three or more parities. 57.4% of the women had no education or an elementary school education.

**Table 1:** Sociodemographic characteristics and mental health (n = 3000).

Characteristics		N (%)
Age (years)	65–69	1000 (33.3)
	70–74	1000 (33.3)
	75–79	1000 (33.3)
	Mean (SD)	71.8 (4.3)
BMI (kg/m^2^)	Underweight (< 18.5)	58 (1.9)
	Normal (≥ 18.5 and < 23)	1127 (37.6)
	Overweight (≥ 23 and < 25)	872 (29.1)
	Obese (≥ 25)	943 (31.4)
	Mean (SD)	23.7 (2.6)
Hysterectomy	No	2677 (89.2)
	Yes	323 (10.8)
Parity	0	109 (3.6)
	1	85 (2.8)
	2	627 (20.9)
	3 or more	2179 (72.6)
Income($/month)	< 2000	1837 (61.2)
	2000–3999	880 (29.3)
	≥ 4000	283 (9.4)
Education	Illiterate	428 (14.3)
	Elementary school	1292 (43.1)
	Middle School	815 (27.2)
	High school or more	465 (15.5)
Geriatric Depression Scale	None (0–4)	1574 (52.5)
	Mild (5–9)	1017 (33.9)
	Moderate to severe (10–15)	409 (13.6)
	Mean (SD)	4.9 (3.9)
Perceived Stress Scale	Very low (0–4)	451 (15.0)
	Low (5–8)	1929 (64.3)
	High (9–12)	586 (19.5)
	Very high (13–16)	34 (1.1)
	Mean (SD)	6.8 (2.1)
Rosenberg Self-esteem Scale	High (20–30)	1236 (41.2)
	Medium (10–19)	1735 (57.8)
	Low (0–9)	29 (1.0)
	Mean (SD)	18.5 (3.6)

Among all of the women in our sample, 13.6% had moderate to severe depression defined by a GDS score of 10 or more. The mean score for PSS-4 was 6.8, with a SD of 2.1. High stress levels (PSS-4 score > 9) were reported by 620 (20.7%) respondents. 1.0% of the women reported low self-esteem defined by an RSES score of 9 or less.

Table 2 lists the prevalence of UI and mental health state according to the individual types of UI. The prevalences of SUI and UUI were 45.7% and 39.6%. The prevalence of incontinent women was 52.2% regardless of the type of UI. The prevalence of MUI was 33.1%.

**Table 2:** Depression, stress, and self-esteem levels according to type of urinary incontinence.

Total	Stress Incontinence			Urge Incontinence			Mixed Incontinence			
	No (%)	Yes (%)	*P *value	No (%)	Yes (%)	*P *value	None (%)	Any UI (%)	Mixed UI (%)	*P* value
	1628 (54.3)	1372 (45.7)		1812 (60.4)	1188 (39.6)		1434 (47.8)	572 (19.1)	994 (33.1)	
**Geriatric Depression Scale**										
None (0-4)	989 (60.8)	585 (42.6)	<.0001	1081 (59.7)	493 (41.5)	<.0001	894 (62.3)	282 (49.3)	398 (40.0)	<.0001
Mild (5-9)	486 (29.9)	531 (38.7)		560 (30.9)	457 (38.5)		407 (28.4)	232 (40.6)	378 (38.0)	
Moderate to severe (10-15)	153 (9.4)	256 (18.7)		171 (9.4)	238 (20.0)		133 (9.3)	58 (10.1)	218 (21.9)	
Mean (SD)	4.0 (3.5)	5.8 (4.0)	<.0001	4.1 (3.6)	6.0 (6.0)	<.0001	3.9 (3.6)	4.8 (3.4)	6.2 (4.2)	<.0001
**Perceived Stress Scale**										
Very low (0~4)	311 (19.1)	140 (10.2)	<.0001	355 (19.6)	96 (8.1)	<.0001	296 (20.6)	74 (12.9)	81 (8.2)	<.0001
Low (5~8)	1059 (65.1)	870 (63.4)		1166 (64.4)	763 (64.2)		911 (63.5)	403 (70.5)	615 (61.9)	
High/very high (9~16)	258 (15.9)	362 (26.4)		291 (16.1)	329 (27.7)		227 (15.8)	95 (16.6)	298 (30.0)	
Mean (SD)	6.5 (2.1)	7.2 (2.1)	<.0001	6.5 (2.1)	7.3 (2.0)	<.0001	6.5 (2.2)	6.7 (1.9)	7.4 (2.0)	<.0001
**Rosenberg Self-esteem Scale **										
High (20~30)	785 (48.2)	451 (32.9)	<.0001	822 (45.4)	414 (34.9)	<.0001	688 (48.0)	231 (40.4)	317 (31.9)	<.0001
Low or medium (0-19)	843 (51.8)	921 (67.1)		990 (54.6)	774 (65.2)		746 (52.0)	341 (59.6)	677 (68.1)	
Mean (SD)	19.1 (3.5)	17.8 (3.6)	<.0001	18.9 (3.5)	17.8 (3.6)	<.0001	19.1 (3.5)	18.4 (3.5)	17.7(3.6)	<.0001

Depression and stress levels were significantly higher in women with UI than those without UI (p < 0.001). Women with SUI or UUI reported significantly higher depression and stress levels than those without SUI or UUI. Women with SUI or UUI had significantly lower levels of self-esteem according to RSES than those without SUI or UUI. Women with MUI reported a mean score on the GDS of 6.2. Those without any UI showed a mean score on the GDS of 3.9. Women with MUI reported a mean score on the RSES of 17.7, and those without any UI showed a mean score on the GDS of 19.1.

For women with MUI, almost one in five had moderate to severe levels of depression defined by a GDS score of 10 or more and 30.0% had high levels of stress defined by a PSS-4 score of 9 or more. 68.1% reported low to medium levels of self-esteem defined by an RSES score of 19 or less. Among the different types of UI, the lowest levels of self-esteem were seen for MUI.

Significant associations were noted between UI and high levels of depression and stress and a low level of self-esteem (Table 3). After adjustment for age, BMI, parity, hysterectomy, income, and education, UI was still significantly associated with depression, stress, and self-esteem. Women with MUI were likely to have scores of 1.9 points higher on the GDS, 0.8 points higher on the PSS-4, and 1.2 points lower on the RSES than women without UI.

**Table 3:** Associations between urinary incontinence and depression, stress, and self-esteem levels.

Variables	Geriatric Depression Scale		Perceived Stress Scale		Rosenberg Self-esteem Scale	
	Estimate	*P* value	Estimate	*P* value	Estimate	*P* value
**Urinary Incontinency**						
Mixed UI	1.911	< .0001	0.808	< .0001	− 1.17	< .0001
Any UI	0.817	< .0001	0.173	0.089	− 0.642	0.000
None	Ref		Ref		Ref	
Age (years)	0.092	< .0001	0.049	< .0001	− 0.100	< .0001
**BMI (kg/m**^**2**^)						
Underweight (< 18.5)	0.360	0.466	− 0.142	0.606	− 0.213	0.649
Normal (≥ 18.5 and < 23)	0.069	0.671	− 0.045	0.621	0.156	0.313
Overweight (≥ 23 and < 25),	− 0.036	0.829	0.006	0.946	0.291	0.064
Obese (≥ 25)	Ref		Ref		Ref	
**Hysterectomy**						
Yes	0.676	0.002	0.433	0.000	− 0.522	0.012
No	Ref		Ref		Ref	
**Parity**						
3 or more	0.904	0.089	0.021	0.945	0.037	0.941
2	0.361	0.349	− 0.223	0.300	0.406	0.268
1	0.224	0.537	− 0.248	0.221	0.814	0.018
0	Ref		Ref		Ref	
**Income ($/month)**						
< 2000	0.991	< .0001	0.408	0.003	− 1.013	< .0001
2000–3999	0.001	0.997	0.028	0.841	− 0.323	0.176
≥ 4000	Ref		Ref		Ref	
**Education**						
Illiterate	1.058	0.000	0.350	0.021	− 0.172	0.508
Elementary school	0.734	0.001	0.407	0.001	− 0.325	0.116
Middle School	0.526	0.017	0.298	0.015	− 0.490	0.019
High school or more	Ref		Ref		Ref	

Meanwhile, depression, stress, and low self-esteem were also significantly related with age, hysterectomy, income, and education. Depression and stress levels were significantly higher and self-esteem levels were lower in women who had undergone a hysterectomy than those who had not. No significant relationships were noted between mental health and BMI or parity.

## Discussion

In this study, we discovered that UI affects at least one in two older Korean women (52.2%). The prevalences of SUI, UUI, and MUI were 45.7%, 39.6%, and 33.1%, respectively. UI was found to be adversely associated with depression, stress, and self-esteem: women with UI reported significantly higher levels of depression and stress and lower levels of self-esteem than those without UI. Women with MUI reported significantly greater impairment than the women with SUI or UUI. The observed associations persisted after adjusting for age, BMI, parity, hysterectomy, income, and education. Also, women with a hysterectomy reported higher levels of depression and stress and lower self-esteem. This indicates that women who have undergone or will undergo a hysterectomy may require psychological care^11^.

UI has been shown to be bothersome to patients and to negatively affect many aspects of life, including sleep, urinary tract infections, falls, nontraumatic fracture, depression, relationships, work productivity, and self-esteem^2,9,12,13^. However, many older individuals view their UI as a normal feature of age, and this belief may contribute to delays in seeking treatment^14,15^. Despite high rates and its adverse effects on health, UI is underreported by women and, therefore, infrequently recognized by clinicians. Indeed, research has indicated that less than half undergo evaluation or treatment for this burdensome condition^16^. Accordingly, greater awareness of UI as a treatable condition and that it is not a normal part of ageing is needed among the population and health professionals.

Although UI is not a life-threatening condition, it has the potential to have a negative impact on the psychological health of women and to reduce quality of daily living, thereby resulting in a higher predisposition to stress^17^. Incontinence women produces marked loss of self-esteem, depression, loss of independence, and a profound stigma. Social withdrawal is associated with the anxiety related to becoming incontinent in public and the possibility that others may find out, rather than distress related to the leakage of urine itself^18^.

The association between psychiatric illness and incontinence is thought to be multidirectional. Abnormality in serotonin is a plausible explanation for both depression and incontinence development. Involuntary leakage of urine can be bothersome, lead to anxiety, and subsequently contribute to social isolation and depression development^19^. In addition, increased sympathetic nervous system activity associated with depression may increase circulating levels of cortisol and catecholamines and, consequently, lead to physiologic changes in the bladder and UI^2^. The combined impact of UI and depression exceeds the impact of either condition alone. Accordingly, clinician must be prepared to discuss all aspects of women’s lives in order to ascertain all their concerns.

Previously, in the assessment of bladder problems and the treatment thereof, clinicians have focused on objective measures of bladder function, notably urodynamic parameters^18^, and reportedly, physicians are often unaware of the social stigma and psychological problems associated with UI. Accordingly, researchers have recommended routine screening for these psychiatric conditions^20^. Screening would likely have a positive impact on self‐esteem, perception of symptoms, and satisfaction with treatment and would likely contribute to an overall perceived improvement in quality of life. Moreover, early intervention may reduce symptom progression, improve immediate and long-term quality of life, and limit the need for more complex and costly treatment. To maximize the opportunity for successful treatment, routine mental health screening and close collaboration with mental health professionals are critical^13,21^.

Our study has several limitations. First, since UI symptoms were self-reported, it is likely that we may have over- or underestimated the prevalence. However, the validity of self-reported UI, compared with clinical diagnosis, has been established in previous research^22^. Second, the reported prevalences of UI vary considerably in different studies, with differing definitions for UI having been used: prevalence was estimated from frequency of UI abstracted as daily, weekly, or monthly episodes of urine. This could limit our ability to compare. This study was a cross‐sectional design, and no causality or direction of association can be established. Finally, gynecological conditions such as prolapse of the uterus, surgery involving the pelvic floor as well as medical conditions such as diabetes mellitus are some of the key risk factors for UI^23^. Although we considered a wide variety of variables for adjustment in the multivariable models, we cannot rule out the presence of an unmeasured confounder. Despite these limitations, we investigated incontinence among older women from a nationally representative cross-sectional survey. UI is a common cross-cultural condition. Ethnicity can affect the association between UI and mental health^24^. Therefore, a study in Korean women is essential to understand ethnic differences in the emotional significance of UI.

Altogether, our results highlight the effect of incontinence on mental health and provide an evidence base for the evaluation of mental health in older women with incontinence. We propose that the prioritization of UI detection and the identification of psychological factors associated therewith may help improve the diagnosis and management of UI and potentially yield significant economic and psychosocial benefits.

## Methods

### Data and study population

Data were obtained from the 2016 Korean Study of Women’s Health Related Issues (K-Stori). We conducted K-Stori, a nationwide cross-sectional survey targeting Korean women aged 14–79 year to investigate current interests into health among the general population of women in Korea^25^. Therein, stratified multistage random sampling was applied to select 3000 women in each life cycle (adolescence, 14–17 years; childbearing, 19–44 years; pregnancy and postpartum, 19–44 years; perimenopause, 45–64 years; and older adulthood, 65–79 years). Of the 15,000 women who participated in the K-Stori survey, UI was investigated only in women in older adulthood stages. A total of 3,000 women between 65 and 79 years were the final study subjects. Trained interviewers contacted candidate participants by going door-to-door to assess their study eligibility. All eligible participants in the house were then surveyed using a standardized questionnaire through face-to-face interviews after obtaining informed consent. The survey response rate was 40.4%. Details on K-Stori have been described elsewhere^25^.

### Dependent variables

Our dependent variables of interest were defined as depression, stress, and self-esteem. Depression symptoms were assessed using the 15-item Geriatric Depression Scale (GDS-15), which is a short form of GDS used to screen, diagnose, and evaluate depression in older adult individuals. Previous studies have shown that GDS-15 can be used to discriminate between depressive and non-depressive states^26–28^. GDS-15 has been translated into Korean^29,30^. GDS-15 scores range from 0–15: 0–4, none; 5–9, mild; and 10–15, moderate to severe depression. A score of > 5 suggests the presence of depression, and a score ≥ 10 is almost always indicative of depression^30^.

The Perceived Stress Scale (PSS) is a widely used psychological instruments for measuring nonspecific perceived stress. The four‐item version of the PSS (PSS‐4) is a brief tool, with items selected from a pool of 14 items included in the original version of PSS^31–34^. The 4-item version (PSS-4) has two negative and two positive items. Subject responses are measured on a five-point scale (0 = never, 1 = almost never, 2 = sometimes, 3 = fairly often, 4 = very often). Total scores range from 0 to 16, with higher scores indicating higher levels of perceived stress: 0–4, very low; 5–8, low; 9–12, high; and 13–16, very high^35^. We used Korean versions of PSS-4^33^.

Self-esteem is defined as a positive or negative attitude toward oneself. It can also be defined as an individual's sense of self-worth. The Rosenberg Self-esteem Scale (RSES) is a standardized resource widely known and applied in clinical practice and research^36^. RSES consists of 10 items known to be positively correlated with self-esteem. The highest possible total is 30, with results presented in three levels: low (0–9), medium (10–19), and high (20–30) self-esteem^37^.

### Independent variables

Our primary independent variable was UI. SUI was defined as a complaint of involuntary leakage on effort or exertion or on sneezing or coughing. SUI was assessed with the question “During the past week, did you lose urine on coughing, sneezing, or exercising?” Responses were scored on a dichotomous scale (i.e., “yes” or “no”)^38,39^. UUI was defined by a positive response to the question ‘‘During the past week, have you had any involuntary leakage accompanied by or immediately preceded by urgency?’’^39,40^. Patients with symptoms of both disorders may be afflicted by MUI^39^.

Age, body mass index (BMI), parity, hysterectomy, income, and education were considered as covariates. Women were categorized into 5-year interval age groups: 65–69, 70–74, or 75–79 years. BMI was calculated using self-reported height and weight data, and participants were categorized according to criteria for Asians or generally applied definitions for the Korean population (5, 36, 37) using BMI cut offs of < 18.5 kg/m^2^ (underweight), < 23 kg/m^2^ (normal), ≥ 23 kg/m^2^ (overweight), and ≥ 25 kg/m^2^ (obese)^41^. Education level was categorized as ≤ elementary school, middle school, high school, or college or more. Household income was categorized as < $2000/month, $2000–3999/month, or ≥ $4000/month.

### Statistical analysis

We investigated the general characteristics of the study population and the prevalence rates of SUI, UUI, and MUI. We compared depression, stress, and self-esteem levels with the status of UI using χ2 square tests. We applied multiple linear regression models to analyze associations with depression, stress, and self-esteem levels in relation to UI type. Estimates were adjusted for the following socio-demographic characteristics: age, BMI, hysterectomy, parity, income, and education.

All analyses were conducted using SAS 9.3 (SAS Institute, Cary, NC, USA). *P* values less than 0.05 were considered statistically significant, and all reported *P* values were two sided. All subjects participated voluntarily and provided informed consent. Informed consent was obtained from their legal guardians for illiterate participants. The study was approved by the institutional review board of the National Cancer Center, Korea (NCC2016-0062). This study was performed in accordance with the relevant guidelines and regulations.
